# Short-term effects of transcutaneous auricular vagus nerve stimulation on T-wave alternans in people with focal epilepsy – An exploratory pilot study

**DOI:** 10.1016/j.ebr.2024.100657

**Published:** 2024-03-05

**Authors:** Jan Pukropski, Jan Baumann, Arthur Jordan, Marcel Bausch, Randi von Wrede, Rainer Surges

**Affiliations:** Department of Epileptology, University Hospital Bonn, Germany

**Keywords:** T-wave alternans, Chronic epilepsy, Vagus nerve stimulation, Neuromodulation, Sudden cardiac death, Cardioprotective therapy

## Abstract

High levels of T-wave alternans (TWA) are linked to an increased risk of sudden cardiac death. People with epilepsy display elevated TWA levels that are decreased by chronic vagus nerve stimulation via implanted devices after 2–4 weeks or later. Our objective was to explore short-term effects of transcutaneous auricular vagus nerve stimulation (tVNS) on TWA. Five patients (3 female) with focal epilepsy undergoing video-EEG monitoring were included. TWA levels were determined using a one-channel modified lead I ECG via an open-source TWA-algorithm on two consecutive days, 1 h before, during and after tVNS via the left auricle. Data are given as mean ± SE. Mean TWA at baseline was 3.8 ± 0.4 µV and 3.0 ± 0.6 µV during stimulation on day 2. Stimulations on the second day were associated with TWA reductions by 22 ± 13 % that exceeded stimulation effects on the first day relative to baseline (p < 0.05). Linear mixed-models revealed effects of both stimulation (p < 0.05) and stimulation number (p < 0.005). Normalized TWA showed reproducible peak reductions at both days within 35 min after the initiation of tVNS (p < 0.05). Our observations suggest that tVNS has short-term effects on TWA, supporting the notion that vagus nerve stimulation has a beneficial impact on electrical cardiac properties.

## Highlights

• tVNS was performed on two consecutive days in 5 subjects with focal epilepsy.
• tVNS reduced TWA levels already shortly after initiation of the stimulation.
• tVNS may have a protective impact on electrical heart properties.

## 1 Introduction

Epilepsy is a common chronic neurological disorder affecting over 50 million people worldwide [1]. More than 25 antiseizure medications (ASM) for seizure control are available, but approximately one third of people with epilepsy (PWE) still suffer from recurrent seizures despite continuous ASM [1]. PWE have a nearly threefold elevated risk of dying from sudden cardiac death (SCD) compared to the general population [2], [3]. Importantly, signs of cardiac damage and electrical instability are increasingly recognized in postmortem analysis and ECG studies of living PWE, which has recently led to the concept of the ‘epileptic heart’ [2], [4].

One potential feature to reflect properties of the ‘epileptic heart’ is T-wave alternans (TWA), which assesses the beat-to-beat fluctuation in the course of the ST segment in the electrocardiogram. Higher levels of TWA were shown to be associated with an increased SCD risk in patients with heart failure; similarly elevated TWA levels were also described in PWE in the interictal and early postictal period [5], [6]. Invasive vagus nerve stimulation (iVNS) is an established antiseizure treatment [7], [8]. Importantly, iVNS was also shown to reduce TWA levels in PWE two to eight weeks after titration of implanted VNS device [6], [9].

Transcutaneous auricular vagus nerve stimulation (tVNS) allows non-invasive stimulation of the vagus nerve in the area of the left auricle and has been shown to improve seizure control [10]. Here, we explored, in analogy to iVNS, whether tVNS exerts a measurable effect on TWA.

## 2 Methods

### Patient selection

We recruited five subjects with pharmacoresistant epilepsy undergoing Video-EEG with one-channel electrocardiogram at our epilepsy monitoring unit in May 2023. Inclusion criteria were the diagnosis of a pharmacoresistant epilepsy with clinical indication for video-EEG-monitoring on two consecutive days. Exclusion criteria were seizures occurrence in the last 24 h before video-EEG-monitoring, recent or previous neurostimulation by iVNS or deep brain stimulation and mental disability. All subjects signed informed consent before starting the tVNS. The study protocol had been approved by the ethics committee of the Rheinische Friedrich-Wilhelms-Universität Bonn (No. 442/19) and is in accordance with relevant guidelines and regulations.

### 2.2 Transcutaneous auricular vagus nerve stimulation

Stimulation was performed with two hemispheric titanium electrodes of a NEMOS device (tVNS Technologies GmbH, Erlangen, Germany) in the region of auricular branch of the vagus nerve (cymba conchae) on two consecutive days at the same time of the day in the afternoon (2 pm) for 1 h. The stimulation current of 2 mA was achieved by titration over a few seconds, and care was taken to ensure that participants did not feel pain from the stimulation. The standard parameter of stimulation (biphasic waveform, pulse duration 20 s, pulse pause 30 s, pulse frequency 25 Hz) were identical in all subjects. We have retrospectively checked that ASM was not changed during the two days of stimulation.

### 2.3 ECG and EEG recordings

Electrical heart properties were determined using the standard one-channel electrocardiogram during video-EEG monitoring with adhesive electrodes placed below the two clavicles. The lead is comparable to lead I of the Einthoven triangle. On both days, recordings were performed over three hours – one hour before (baseline), one hour during (stimulation) and one hour after tVNS (post stimulation). During this 3-hour block, subjects were awake and relaxed in lying position. In addition, we also recorded electroencephalograms (EEG) according to the 10–20 system. We visually reviewed all EEG recordings to rule out seizures or subclinical seizure patterns and to detect regular VNS stimulation activity in the EEG electrodes near the left auricle as an indication of its proper use.

### 2.4 ECG analysis

TWA was calculated with the open-source algorithm by Khaustov and Cliffordusing a Matlab implementation [11], [12]. Each 15-second-window of the input ECG signal was split into sets of even and odd beats (Supplementary Fig. 1, upper panels). For each set, the mean waveform was calculated and the maximum difference between the two sets in the interval from J-point to the end of the T-wave was returned as TWA for this window. Additionally, automated detection of artifacts has been implemented so that the corresponding TWA value could be removed from the set if necessary. To this end, each of the ST-segments within each set was compared with every other segment within the set by averaging the absolute difference, resulting in a distance matrix for each set. If a single distance value was above a predefined threshold of 100 µV, the current window’s TWA was flagged as an artifact (Supplementary Fig. 1). The mean TWA during the one-hour blocks (baseline, stimulation, post stimulation) is given as the average TWA value for each patient.

### 2.5 Statistical methods

All analyses were performed in Matlab R2021a. TWA and their reductions are reported as means ± standard error or mean z-values ± standard error relative to baseline. The effect of tVNS on TWA and its interaction with stimulation number (day) were fit with a linear mixed effects model (fitlme). Stimulation effects within subjects were modeled as random intercepts and slopes. In post-hoc analyses, reductions of TWA or z-values during stimulation were quantified by one-sided Mann–Whitney U (all TWA per subject) or Wilcoxon signed rank tests (session-averages) without correction for multiple comparisons. P values < 0.05 were considered statistically significant.

## 3 Results

All 5 subjects (3 females) had focal epilepsy that did not respond to at least two ASMs. The participants’ age was 31.2 ± 9.4 years, the epilepsy duration amounted to 21.6 ± 13.5 years. In 4 subjects, EEG showed interictal epileptiform potentials over the temporal regions (2 on left, 2 on right side). The etiology was unknown in 3 subjects, in one patient GAD65-associated autoimmune epilepsy, and one patient suffered from structural damage following meningoencephalitis in the childhood (with unknown germ). All subjects had normofrequent sinus rhythm and no cardiac complaints or known heart diseases.

**Fig. 1:**
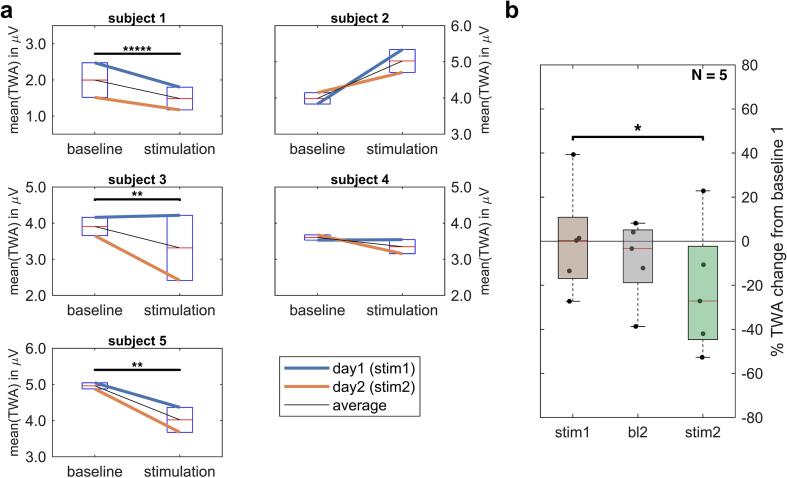
TVNS decreases twa levels with cumulative effects. **a.** Boxplots (Q1, median, Q3; whisker: points within ± 1.5 IQR) of mean TWA for all 5 subjects for baseline versus stimulation (each approximately 60 min) as a function of the stimulation number/day. **b.** Boxplots depicting percent TWA reductions during stimulation 1/2 (STIM1/2) or baseline 2 (BL2) all relative to baseline 1 (BL1). *p < 0.05.

Technically correct stimulation was checked by visual observation of expected artifacts in the EEG upon tVNS in all 5 subjects. No seizures occurred during the ECG recordings. Mean (average) TWA of all patients at baseline on day 1 was 3.8 ± 0.4 µV and 3.0 ± 0.6 µV during stimulation on day 2. Mixed-effects linear models indicated significant short-term effects of tVNS on TWA (p < 0.05) and interactions between stimulation and stimulation number per day (p < 0.005) (Fig. 1a). Four subjects displayed lower TWA levels averaged across both stimulations compared to baseline. Stimulation-induced reductions in the distribution of all TWA were significant for subjects 1, 3 and 5 (one-sided Mann–Whitney U tests). In contrast, subject 2 had higher TWA levels upon tVNS.

**Fig. 2:**
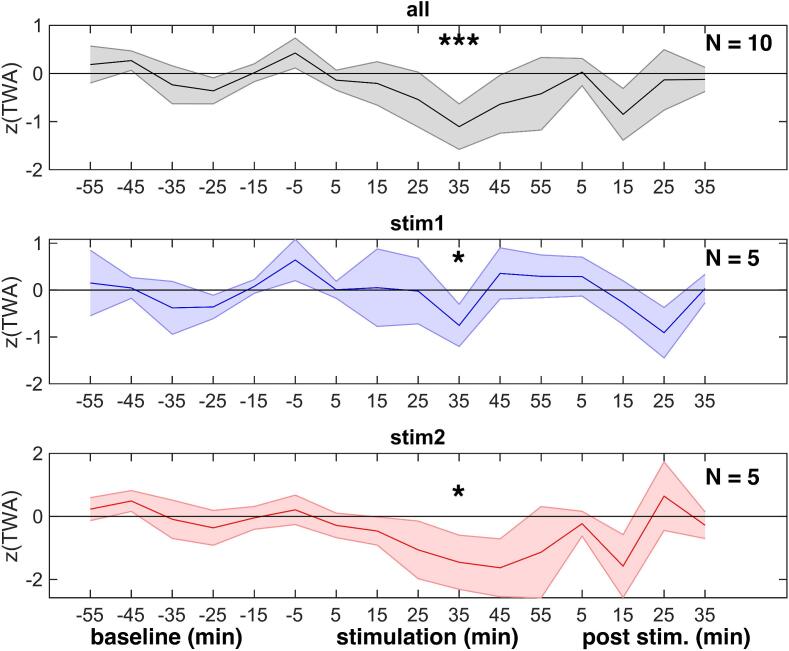
Tvns stimulations are associated with delayed, reproducible reductions in normalized twa. z-values of binned (10 min) TWA at different times relative to baseline, stimulation or post stimulation, all normalized to baseline for each session. *p < 0.05; ***p < 0.001.

Overall, reductions were stronger on day 2 (Fig. 1b, in green), particularly in subjects 3 and 5. Stimulations on the second day were associated with reductions of TWA by 22 ± 13 % relative to baseline on the first day (p < 0.1), and were significantly higher than stimulation effects on the first day relative to its baseline (p < 0.05). Averaged across the whole stimulation period, there were no TWA reductions on day 1 (Fig. 1b). TWA normalized to Z-values, however, showed reproducible reduction-peaks 35 min after the initiation of stimulation on day 1 and 2 (p < 0.05), and also for pooled data of both days (p < 0.001) (Fig. 2). This peak reduction was more substantial and prolonged on day 2. Visually, normalized TWA decreased after approximately 20 min of stimulation and returned to baseline TWA at the end of stimulation.

## 4 Discussion

The elevated risk of SCD, possibly caused by alterations recently labelled as ‘epileptic heart’ [4], underscores the need for novel cardioprotective treatment options in PWE. TWA may serve as a surrogate marker of the ‘epileptic heart’. Potential cardioprotective effects of VNS via reduction of TWA have been reported, but only for implanted devices and after stimulation for several weeks.

We employed auricular tVNS, a method by which stimulation-dependent side effects of iVNS such as cough and voice alteration are absent [10]. This in turn allowed a rapid titration of the stimulation current and enabled us to investigate possible short-term effects on TWA properties. We found evidence for early stimulation effects in 4 of the 5 subjects. Moreover, stimulation-induced reductions in the distribution of all TWA levels were significant in 3 subjects. Similar to our results, Schomer and colleagues showed individual response of iVNS after stimulation over 7.6 weeks (range: 1–14 weeks) in 6 of 9 patients [9]. No increase in TWA was observed in any of these 6 patients, but the stimulation parameters as well as the stimulation current varied between patients. Compared to our short-term mean TWA reduction from 3.8 to 3.0 µV (TWA reduction by 21 %), their maximum reduction in TWA amounted to 35–40 %, depending on the ECG lead (V1, V2, aVF). This is consistent with a presumed cumulative effect of repetitive stimulation.

The mechanisms by which iVNS reduces TWA levels are not well understood. However, both improved seizure control and direct effects via efferents and afferents of the autonomic nervous system may play a role [6], [9]. The auricular branch of vagus nerve in the region of the cymba conchae is innervated only by vagal afferent fibers and results in activation of vagal projections such as the nucleus tractus solitarii and the locus coeruleus in the central nervous system [13], [14]. This suggests that the effects observed in our small scope study are mediated via the central nervous system, e.g. by short term effects with rather early onset in the brain stem networks.

Our exploratory pilot study comes with the limitations of a small sample size and the lack of a control group. Since we consider short time periods (minutes to hours) with subtle and delayed effects of tVNS after start of stimulation, we used mean TWA values for each one-hour block for continuous monitoring. In contrast to previous iVNS-studies in people with epilepsy, we did not use maximum TWA values of the Modified Moving Average (MMA) method, which can vary within several minutes and could possibly reach high values at the beginning of tVNS stimulation without stimulation effects on TWA. Also we used an open-source TWA algorithm and a modified ECG lead I. Therefore, a direct comparison between our TWA values and TWA values of previous iVNS studies is hampered [5], [6], [9]. Mean (average) TWA was also applied in former studies for risk stratification in patients with chronic heart failure [15], [16]. Our TWA values are more comparable to the smaller average TWA values of the spectral method for TWA analysis, whereas the maximum TWA values of the Modified Moving Average (MMA) method are larger by a factor of 4 to 10 [17]. However, our results provide proof-of-concept evidence that tVNS exerts measurable effects with early onset on TWA under controlled conditions and support the notion of VNS-related cardioprotective effects. Future trials are planned to investigate these effects in larger group and at a longer time scale. The influence of temporal arrangement and number of stimulation blocks in evaluating a cumulative effect as a possible sign of successful neuromodulation should also be further explored.

In conclusion, tVNS significantly reduces TWA already shortly after initiation of the stimulation and possibly exerts an early cumulative effect of repeated stimulations as a possible sign of effective neuromodulation in the central nervous system. Our results support the notion that vagus nerve stimulation may have a beneficial impact on electrical heart properties, which may reduce the cardiac risks in patients with chronic epilepsy.

## Ethical statement

All subjects signed informed consent before starting the tVNS. The study protocol had been approved by the ethics committee of the Rheinische Friedrich-Wilhelms-Universität Bonn (No. 442/19) and is in accordance with relevant guidelines and regulations.

## CRediT authorship contribution statement

**Jan Pukropski:** Writing – original draft, Methodology, Investigation, Conceptualization. **Jan Baumann:** Writing – original draft, Methodology, Investigation, Conceptualization. **Arthur Jordan:** Writing – review & editing, Software, Resources. **Marcel Bausch:** Writing – review & editing, Visualization, Validation, Investigation. **Randi von Wrede:** Writing – review & editing, Resources. **Rainer Surges:** Writing – review & editing, Writing – original draft, Supervision, Conceptualization.

## Declaration of competing interest

The authors declare the following financial interests/personal relationships which may be considered as potential competing interests: RS has received personal fees as speaker or for serving on advisory boards from Angelini, Arvelle, Bial, Desitin, Eisai, Jazz Pharmaceuticals Germany GmbH, Janssen-Cilag GmbH, LivaNova, LivAssured B.V., Novartis, Precisis GmbH, Rapport Therapeutics, Tabuk Pharmaceuticals, UCB Pharma, UNEEG, and Zogenix. These activities were not related to the content of this manuscript. RvW has received personal fees as speaker, for travel support, for attending meetings or for serving on advisory boards from Angelini, Arvelle, Bial, Eisai, Jazz Pharmaceuticals Germany GmbH/GW Pharma, UCB Pharma, Xenon Pharmaceuticals. These activities were not related to the content of this manuscript. JP, JB, AJ, MB have no interests to disclose.
