# Supplementary material for: Short-term effects of transcutaneous auricular vagus nerve stimulation on T-wave alternans in people with focal epilepsy – An exploratory pilot study
Source: Epilepsy Behav Rep. 2024 Mar 5;26:100657. doi: 10.1016/j.ebr.2024.100657 (PMC10940126; doi:10.1016/j.ebr.2024.100657)
Supplement: Supplementary data 1 [file mmc1.docx]

**Supplementary figure 1**

**
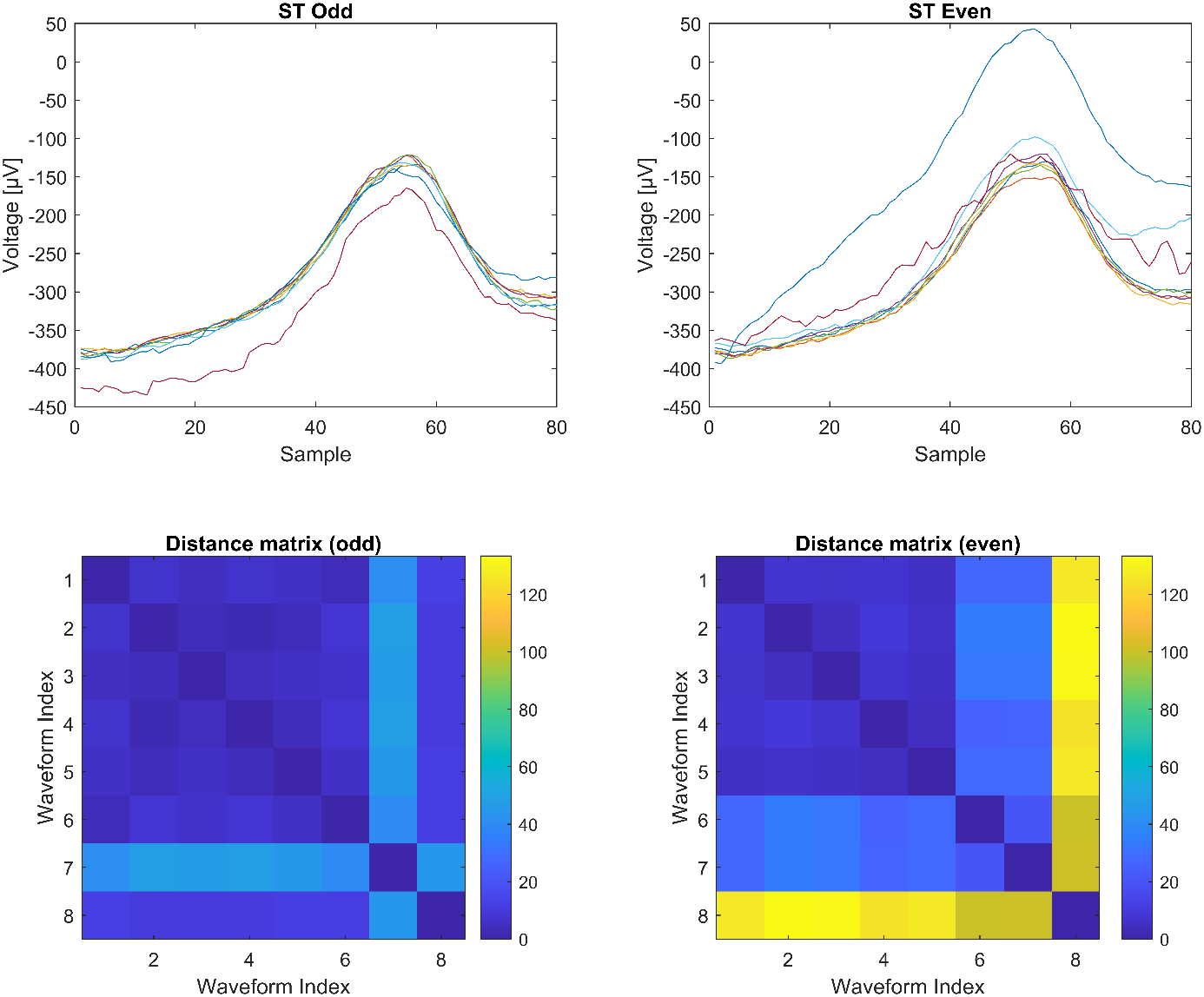
**

*Supplementary figure 1.* Example of artifacts in both waveform sets with corresponding distance matrices. Upper panels with plots of odd and even ST segments. The upper blue line of ST even waveforms has been flagged as an artifact above the threshold (corresponding yellow boxes in the lower distance matrix). Lower panels show corresponding 8x8 matrix with color-coded absolute mean differences between each pair of all 8 waveforms in a 15-second window. We used a threshold of 100 µV for detection of artifacts.
